# Performance analysis of DTC-SVM in a complete traction motor control mechanism for a battery electric vehicle

**DOI:** 10.1016/j.heliyon.2022.e09265

**Published:** 2022-04-12

**Authors:** Matthew Liam De Klerk, Akshay Kumar Saha

**Affiliations:** Discipline of Electrical, Electronics & Computer Engineering, University of KwaZulu-Natal, 238 Mazisi Kunene Road, Durban, 4041, South Africa

**Keywords:** Direct torque control (DTC), DTC-SVM, Electric vehicles, Field-weakening control, Sensorless control, Traction motor control system

## Abstract

The transport sector is essential for socio-economic growth; however, the sector contributes a large portion of global carbon dioxide emissions. Consequently, electric vehicles have received increasing attention from various stakeholders in order to provide a solution to the current environmental impact of the transport industry. Current literature on electric vehicle powertrains show that electric vehicles are complex systems, with one of the most essential subsystems being the traction motor control mechanism. As a result, the objective of this paper is to add to the research conducted in the field of electric vehicle powertrains by carrying out comprehensive investigations into the suitability and performance of space vector modulation based direct torque control in the traction motor control system of an electric vehicle with a complete drive system. Initially, a conventional direct torque control mechanism was implemented for control of the traction motor system. The results of the investigation into implementation of conventional direct torque control highlighted the expected issues associated with the mechanism. Implementation of improvements to the conventional direct torque control model, such as the use of a space vector modulation based direct torque control model with closed loop torque control, field-weakening and sensorless control produced significantly favorable results, providing decreased torque and current ripples, operation over a wide speed range, and accurate speed control without the need for mechanical speed sensors. Such results demonstrate that the complete control mechanism investigated is well suited for use in electric vehicle applications.

## 1 Introduction

The operation of the majority of cities and business sectors globally require the use of transport and vehicular travel. Internal combustion engine vehicles (ICEVs) are the most commonly used type of vehicle; however, these vehicles cause tailpipe emissions, generating urban air pollution and contributing to greenhouse gases. Objectives and deadlines relating to the reduction of carbon emissions were provided to all countries by the United Nations [1], [2]. However, due to an increasing population size, urbanization, and socio-economic development, an increase in vehicle usage, and the resulting emissions have been observed [3], [4]. ICEVs emit greenhouse gases which include nitrous oxide (N_2_O), methane (CH_4_), carbon dioxide (CO_2_), and various other pollutants [3]. 23-26% of global CO_2_ emissions, and 74% of the on-road CO_2_ emissions were as a result of the transport sector in 2004 and 2007 respectively [3], [4]. The major reliance on transport for economic growth is causing long-term damage to the environment. Additionally, with the increasing consumption of fossil fuels, the future depletion of the non-renewable resources is an undeniable concern [4]. Measures must be taken in order to reduce the environmental effects of the transport industry on both the environment and the depletion of the global natural resources [4]. One of the major solutions to the current environmental issues faced is the consideration and development of Hybrid electric vehicles (HEVs), fuel cell electric vehicles (FCEVs) and battery, or pure electric vehicles (BEVs) [1], [5], [6].

Hybrid electric vehicles are a combination of conventional internal combustion engine technology, and electric power technology [1], [6]. HEVs reduce the consumption of fuel, as it is no longer the only source of energy; however, HEVs do still have greenhouse gas emissions due to the fuel used [1]. Fuel cell electric vehicles make use of fuel cells in order to generate electricity, in which hydrogen is used as an input [1], [5]. Lastly, battery electric vehicles are pure electric vehicles which use batteries as a source of power. There are considerable benefits in the use of this classification of vehicle, which include zero gas or pollutant emissions, high efficiency, independence from petroleum, and quiet and smooth operation with low noise pollution [1], [3], [6], [7]. However, one of the disadvantages currently experienced is the driving range in pure BEVs [1], [6].

The performance of electric vehicles can be improved through the implementation of various mechanical design concepts. However, further efficiency and range improvements can also be achieved through EV powertrain optimization, with focus on EV powertrain electrification. One aspect in such optimization includes enhancement of EV driving range through the use of highly efficient electric motors [2], [8]. Instant and high torques at low-speed operation can be achieved with the application of electric propulsion, and as a result, the requirements associated with urban driving conditions can be met with EV technology [2], [9].

As a result, a key component in the EV powertrain is the traction motor control system. Field oriented control (FOC) and direct torque control (DTC) are the two commonly utilized control techniques. This paper aims to present a study on the performance of DTC in electric vehicle applications, through the development of a complete traction motor control system. Various other research works have been conducted which investigate the traction motor control system of an EV. The authors in [10] make use of ANN DTC in an effort to minimize the issues present with conventional DTC and improve the performance of the EV. Methods of improving the drivetrain efficiency through novel DTC schemes are presented in a number of cases. One of these is presented by the researchers in [11], who utilize the IM loss model to develop a variable flux reference selection technique. A similar technique is presented by the researchers in [12], who utilize a stator current minimization technique to determine the optimal stator flux reference value. The performance and efficiency are further improved with the implementation of a variable DC link voltage system. The authors in [13] present a comprehensive comparison of the performance of CDTC and DTC-SVM with closed loop torque and flux control. Efficiency is a major focus of the comparison conducted, with efficiency optimization techniques implemented in both control schemes compared. Research surrounding the improvement of vehicle rideability is conducted in [14], in which the authors present a modified torque hysteresis controller which allows for torque ripple reduction in conventional DTC. Finally, other works are presented by the authors in [15] and [16]. A sliding-mode based DTC scheme for the control of a four in-wheel drive EV is presented in [15], whereas the authors in [16] attempt to minimize the integral time-weighted absolute error (ITAE) in a CDTC scheme with the implementation of a fractional-order PI controller.

DTC presents promising results in various scenarios and studies analyzed in literature. Such studies make DTC based mechanisms worthy of attention and investigation. However, many of the aforementioned studies that have been presented consider small scale models, in which experimental testing forms the basis of the conclusions made. Even in cases in which simulation studies are presented, low power rating motors and systems are investigated. As a result, this paper aims to provide a study on the performance of a complete traction motor drive in which DTC-SVM is utilized. To the best of our knowledge, a study which investigates motor sizing through parameter matching, while also considering improvements to the control mechanism, including ripple reduction, field-weakening control, and sensorless control has not yet been reported. Investigations of all such aspects are conducted in this paper, while comprehensively considering the performance of all sections of the drive system, simulated over the entire speed range required, at realistic torque loads. As a result, the study provided is extremely comprehensive, considering the vast majority of sub-systems required in an EV traction motor drive. It is essential to understand the theoretical performance of control mechanisms in appropriately scaled systems, which are applicable for use in urban and highway driving conditions.

Section 2 of this article provides the EV parameters used in the study. A comprehensive parameter matching stage is discussed in section 3, with section 4 and section 5 discussing the conventional DTC model implemented and the results obtained. Improvements to the DTC model, which include implementation of space vector modulation based direct torque control (DTC-SVM) with closed-loop torque control, field-weakening control and sensorless control are discussed in sections 6 to 11. Finally, section 12 provides a conclusion on the findings of the paper.

## 2 Electric vehicle parameters

Table 1 shows the primary parameters and Table 2 the dynamic specifications of the prototype vehicle being investigated in this paper. The primary parameters and specifications are based on current literature in which designs were carried out, as well as four/five-seater electric vehicles that are currently being manufactured and are available [7], [17], [18], [19].

**Table 1:** Primary Parameters.

Primary Parameter	Value	Primary Parameter	Value
Curb mass (kg)	1150	Rolling resistance coefficient	0.015
Gross mass (kg)	1400	Front area (m^2^)	2.300
Rolling radius (m)	0.300	Aerodynamic drag coefficient	0.275

**Table 2:** Dynamic Performance Specifications.

Dynamic Performance Specification	Value
Maximum speed (km/h)	≥130
0-100 km/h acceleration time (s)	≤15
Maximum Gradeability (%)	≥30 (at 20 km/h)

Additionally, the dynamic performance specifications are also based on everyday use in urban areas and highways for example, the maximum highway speed limit is 120 km/h, and a 0-100 km/h acceleration time of less than 15 seconds is acceptable for smaller cars used for transportation [20].

## 3 Electric motor parameter matching

Electric motors are used as traction motors in electric vehicles, enabling the transformation of electrical energy into mechanical energy [7]. As a result of this, the dynamic performance characteristics of an EV are directly determined by the performance of the traction motor, [17]. In order for a suitable traction motor to be selected, various motor parameters, including the rated power, maximum power, base speed, maximum speed, and maximum torque must be determined [7]. Initially, the type of motor being utilized in the study must be selected. Current EV drives can be evaluated in order to determine a suitable motor for the investigation being carried out.

The authors in [3], [21], [22], [23] investigate the advantages and disadvantages of different motors based on various parameters. An evaluation of the motors based on this research is shown in Table 3. The evaluation rates each motor based on various metrics, with each metric carrying a maximum score of 5. Each metric is weighted equally to determine an average rating for each motor out of 5. Currently, induction motor and PMSM based EV drives are the most commonly used, representing the majority of the vehicles available on the market [3], [9], [21]. The current use of such motors in commercially available vehicles corresponds to the evaluation undertaken in Table 3, in which induction motor and PMSM drives were found to be the most favorable. As a result of this comparison, an induction motor was chosen for use in this investigation due to its controllability, reliability, and cost.

**Table 3:** Evaluation of the Motors Used in EV Drivetrains [3], [21], [22], [23].

Parameter	Motor Type				
	DC	IM	SRM	PMM	PM BLDC
Power Density	2.25	3.5	3.5	4.75	5
Efficiency	2.25	3.5	4	4.75	5
Controllability	5	4.5	3	4	4
Reliability	3	5	5	4	4
Maturity	5	4.75	3.75	4.5	4
Cost Level	4	5	4	3	3
Noise Level	3	5	2	5	5
Maintenance	2	4.5	4.5	4.5	5
Speed Range	2.5	4	4.5	4.5	4
Robustness	3	5	4.5	4	4.25
Size and Weight	3	4	4	5	4
**Average**	**3.18**	**4.43**	**3.89**	**4.36**	**4.30**

In order to determine the specifications of the selected motor, the maximum power of the motor must first be determined. The motor peak power must enable it to satisfy various vehicle specifications, which are the maximum velocity of the vehicle, the climbing performance of the vehicle, and the 0-100 km/h acceleration time of the vehicle [7], [17], [18], [24]. The authors in [7], [17], [18], [24] propose a method of parameter matching to allow for the motor specifications to be determined. The proposed method will be utilized for the parameter matching carried out in this investigation. Equation (1) determines the power required for the vehicle to meet its maximum speed specification [7], [17], [18], [24].

(1) $$P_{maxspeed}=\frac{1}{3600\eta_{t}}\left(mgfv_{max}+\frac{C_{D}Av_{max}^{3}}{21.15}\right)$$

Equation (2) determines the power required for the vehicle to meet its climbing performance (gradeability) specification [7], [17], [18], [24].

(2) $$P_{climbing}=\frac{1}{3600\eta_{t}}\left(mgfv_{c}\cos\alpha_{max}+mgv_{c}\sin\alpha_{max}+\frac{C_{D}Av_{c}^{3}}{21.15}\right)$$

Equation (3) determines the power required for the vehicle to meet its 0-100 km/h acceleration time specification [7], [17], [18], [24].

(3) $$P_{acceleration}=\frac{1}{3600t_{m}\eta_{t}}\left(\delta m\times \frac{v_{m}^{2}}{7.2}+mgf\times \frac{v_{m}}{1.5}\times t_{m}+\frac{C_{D}Av_{m}^{3}}{21.15\times 2.5}\times t_{m}\right)$$

Where, in equation (1)-(3); $\eta_{t}$ is the driveline efficiency (measured from the power source to the driven wheels), the vehicle mass (kg) is represented by *m*, *g* represents the gravitational acceleration (9.80 m/s^2^), the front area of the vehicle (m^2^) is denoted by A, *f* represents the tire rolling resistance coefficient., $C_{D}$ is the aerodynamic drag coefficient, the maximum velocity of the vehicle (km/h) is given by $v_{max}$, $\alpha_{max}$ represents the maximum grading angle of the vehicle (rad), $v_{c}$ is the climbing/grading velocity of the vehicle (km/h), *δ* is the rotational inertial factor of the vehicle, $t_{m}$ is the specified 0-100 km/h acceleration time of the vehicle (s), $v_{m}$ is the final acceleration speed (km/h), $\rho_{a}$ is the air density (1.202 kg/m^3^).

As mentioned previously, the maximum power of the motor must be able to meet the power requirements in equations (1)-(3). As a result, the maximum power required from the motor can be given by equation (4) [7], [17], [18], [24].

(4) $$P_{max}\geq \max\left[P_{maxspeed};P_{climbing};P_{acceleration}\right]$$

The rated power specification of the motor can be determined from the maximum power specification and the overload factor of the traction motor used in the vehicle. The authors in [25], [26] provide discussions of the overload capabilities of traction motors in electric vehicles. The ability for the traction motor to be overloaded for short periods of time (to meet the maximum power requirements) allows for a smaller motor to be chosen, translating to lower vehicle power consumption [25], [26]. Based on the discussions carried out by the authors in [7], [18], [25], [26], a maximum overload factor of 1.5 (50% overload) was chosen for this investigation. As a result, the rated power of the traction motor required can be found using equation (5) [7], [17], [18], [24].

(5) $$P_{rated}=\frac{P_{max}}{k}\;and\;P_{rated}>P_{maxspeed}$$

In which, *k* is the overload factor chosen. In addition to the maximum and rated power specifications of the motor, the maximum speed of the motor must also be determined. The maximum speed specification of the motor can be determined through the use of equation (6) [7], [18], [24].

(6) $$n_{max}=\frac{v_{max}i_{0}}{0.377r}$$

Where; $v_{max}$ is the maximum velocity of the vehicle, $i_{0}$ is the gear ratio (utilizing a fixed gear system in the electric vehicle), and *r* is the rolling radius. The gear ratio can be chosen, and is not calculated specifically. However, the ratio has a minimum value which is required in order for the vehicle to meet its gradeability performance specification. This minimum limit is given in equation (7) [7], [17], [18].

(7) $$\frac{r}{\eta_{t}T_{max}}\left(mgfcos\alpha_{max}+mgsin\alpha_{max}+\frac{C_{D}Av_{c}^{2}}{21.15}\right)\leq i_{0}$$

In which, $T_{max}$ is the maximum torque of the motor. All of the other parameters in equation (7) have been previously defined. The maximum torque of the motor can be found using equation (8) [17], [27].

(8) $$T_{max}=\frac{9.55P_{max}}{n}$$

In which, *n* is the rated speed of the motor. Utilizing equations (1)-(8), the required traction motor specifications can be determined. The required specifications are shown in Table 4 and are based on research articles [7], [17], [18], [24]. Notably, the maximum speed required is approximately 2.7 times the base speed of the induction motor. A motor control scheme with field-weakening implemented allows the motor to operate at speeds above its base speed; as a result, the maximum speed requirement calculated is achievable [28]. The specifications of the induction motor selected for use in this investigation, meeting the specifications shown in Table 4, are given in Appendix A, Table A1.

**Table 4:** Required Traction Motor Specifications.

Parameter	Value	Parameter	Value
Maximum Power	55 kW	Maximum Speed	8046 r.p.m.
Motor Overload Factor	∼1.5	Maximum Torque	177.72
Rated Power	37 kW	Gear Ratio	7
Base Speed (Rated Speed)	3000 (2952) r.p.m.		

## 4 Conventional direct torque control

The electric machine and drive system in an EV powertrain are essential for enabling the dynamic performance specifications of the EV to be met. As these are core technologies in the EV powertrain, adequate operation of the vehicle requires a correctly designed and implemented traction motor control circuit [2], [3], [9]. As an induction motor was selected for use in this investigation (from the evaluation carried out in Table 3), there are three control methods that can be considered, which are variable-voltage variable-frequency (VVVF) control, field-oriented control (FOC) and direct torque control (DTC) [3], [9], [29].

The VVVF mechanism is a relatively simple method of control and implementation, which is widely used in various applications found in industry [29]. It is advantageous as it enables the electromagnetic torque capability of the induction motor to be maximised [30]. However, the VVVF method has some disadvantages in EV applications. The torque is not directly controlled, and as a result, the torque control provided in VVVF control is not fast or accurate enough for high-performance EV applications [28], [29], [30]. Additionally, field orientation is not utilized, and the motor status is ignored [30].

Improved dynamic control and a fast torque response, when compared to the VVVF method, can be achieved with the use of FOC. Furthermore, various other parameters can be controlled, which include the amplitude, position and frequency of the space vectors for the voltages, currents and magnetic flux [2], [29], [30]. High speed operation can be achieved with the use of FOC, with sensorless speed control operation also possible in indirect FOC systems [2], [30]. Lower torque ripple can be achieved in FOC schemes when compared to the performance of DTC based systems; however, there are also certain disadvantages associated with the FOC mechanism. The robustness of the control mechanism is impacted as a result of the dependence of FOC schemes on the parameters and speed of the induction motor. Additionally, various calculations and transformations must be carried out during the operation of the control mechanism (on-line), meaning FOC is a computationally intensive control method [2], [28], [30].

Similar performance to that observed in FOC schemes, without the need for intensive on-line coordinate transformations can be achieved with the use of DTC [2], [29], [31]. Additionally, as direct control of the motor torque is possible in DTC, a fast torque response is achieved without the requirement for the feedback current control performed in FOC [2], [29], [31]. Furthermore, realistic urban (frequent starting/stopping and acceleration) and highway (high-speed operation) driving requirements can be met with the use of DTC, with more advanced DTC mechanisms also allowing for sensorless speed control [2], [31]. However, despite the benefits that DTC provides, conventional direct torque control (CDTC) structures suffer from high torque and current ripple, with a slower start-up response also noticeable [29], [30], [31]. High noise levels and challenging control at low speeds are also introduced due to the variable switching frequency present in CDTC mechanisms [2], [30], [31].

However, DTC was preferred for implementation in the traction motor control mechanism being investigated in this paper. DTC allows for fast torque control of an induction motor, without the requirement for heavy on-line computation, as is required in FOC [29], [31]. In addition, certain methods of DTC implementation allow for speed estimation from sensed stator voltages and currents, eliminating the requirement for mechanical speed sensors at the motor shaft, thereby reducing the cost of the traction motor control system [31]. As a result, DTC allows for a reduced size of the motor drive, reduced hardware complexity, improved noise invulnerability, increased reliability, and less maintenance [31]. High speed operation, as well as frequent starting/stopping and acceleration, are performance requirements the EV traction motor should be able to satisfy. Dynamic operation of the motor, as well as robust flux weakening control can be achieved with the use of DTC schemes [2], [31].

**Figure 1:**
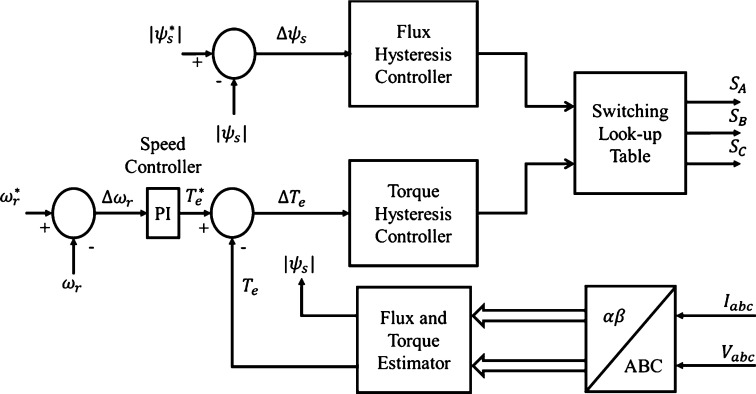
CDTC block diagram (This figure was replicated from [2] - DOI: 10.1109/ACCESS.2021.3110736, using concepts found in [29], [30], [31]).

The block diagram in Fig. 1 shows a conventional DTC system, in which the motor torque and stator flux magnitude are controlled with the use of hysteresis controllers. All induction motor parameters selected for implementation in this investigation are provided in Appendix A, Table A2. The estimator unit included in the control model is used in order to estimate the stator flux and electromagnetic torque of the motor, making CDTC a largely on-line control method [30]. The stator voltage and current in the stationary two-phase reference frame (ds^s^-qs^s^) are required for parameter estimation, and as a result, equation (9) is used to transform the measured stator voltage into the stationary d-q reference frame [29], [30], [31].

(9) $$\left[\begin{array}{c} v_{ds}\\ V_{qs} \end{array}\right]=\frac{2}{3}\left[\begin{array}{ccc} 1\; & -\frac{1}{2}\; & -\frac{1}{2}\\ 0\; & \frac{\sqrt{3}}{2}\; & -\frac{\sqrt{3}}{2} \end{array}\right]\left[\begin{array}{c} v_{as}\\ v_{bs}\\ v_{cs} \end{array}\right]$$

Transformation of the measured stator current into the stationary d-q reference frame can be carried out in a similar manner. Furthermore, the on-line estimation unit uses equations (10)-(13) in order to estimate the electromagnetic torque and stator flux of the motor. The stator flux in the d- and q- axis can be found using equations (10) and (11) respectively [29], [30], [31].

(10) $$\psi_{ds}=\int \left(v_{ds}-R_{s}i_{ds}\right)dt$$

(11) $$\psi_{qs}=\int \left(v_{qs}-R_{s}i_{qs}\right)dt$$

Estimation of the induction motor stator flux magnitude can be performed using equation (12) [29], [30], [31].

(12) $$\left|\psi_{s}\right|=\sqrt{\psi_{ds}^{2}+\psi_{qs}^{2}}$$

Equation (13) enables estimation of the developed electromagnetic torque [29], [30], [31].

(13) $$T_{e}=\frac{3}{2}\frac{P}{2}\left(\psi_{ds}i_{qs}-\psi_{qs}i_{ds}\right)$$

In addition to the stator flux magnitude and the electromagnetic torque, the estimator is also required to estimate the stator flux position in the stationary reference frame. Selection of the inverter switching states requires the position of the stator flux to be known, as it enables the instantaneous flux sector to be found. Calculation of the stator flux position in the stationary reference frame can be carried out using equation (14) [29], [30], [31].

(14) $$\theta_{e}=tan^{-1}\left(\frac{\psi_{qs}}{\psi_{ds}}\right)$$

Where in equations (9)-(14) the applied stator phase voltages (phase a, b and c) are represented by $v_{as},\;v_{bs},\;v_{cs}$ respectively, $v_{ds}$ and $v_{qs}$ represent the transformed stator voltages (stationary d-q reference frame), the stator flux components in the stationary d-q reference frame are given by $\psi_{ds}$ and $\psi_{qs}$, the induction motor stator resistance is indicated by $R_{s}$, $i_{ds}$ and $i_{qs}$ represent the transformed stator currents (stationary d-q reference frame), $\left|\psi_{s}\right|$ is the estimated stator flux magnitude, $T_{e}$ represents developed electromagnetic torque and $\theta_{e}$ represents the stator flux position in the stationary reference frame. Equations (10) and (11) indicate that the stator resistance ($R_{S}$) is required for the electromagnetic torque and stator flux to be estimated; however, equation (15) is found when the stator resistance is neglected [29], [30].

(15) $$\Delta {\overset{\to }{\psi }}_{s}={\overset{\to }{V}}_{s}\Delta t$$

**Figure 2:**
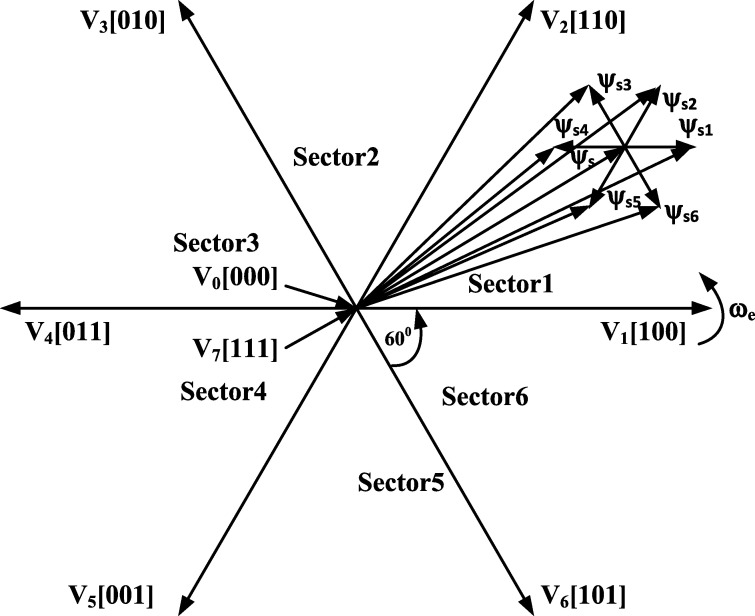
Inverter output voltage space vectors and corresponding stator flux variations for Δ*t* (This figure was replicated from [2] - DOI: 10.1109/ACCESS.2021.3110736, using concepts found in [29], [30]).

**Figure 3:**
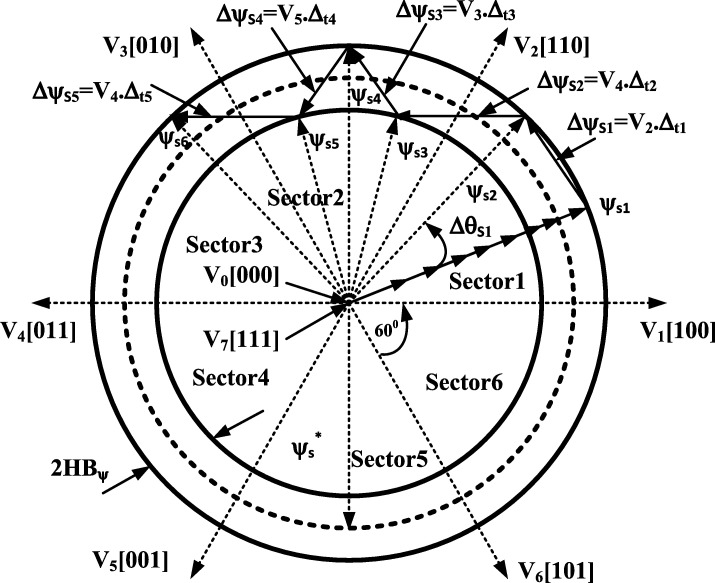
Stator flux trajectory vector in conventional direct torque control (This figure was replicated from [2] - DOI: 10.1109/ACCESS.2021.3110736, using concepts found in [29], [30]).

This simplified equation indicates that a specific stator voltage (${\overset{\to }{V}}_{s}$) applied for a period of time enables the stator flux of the induction motor to be changed. Each space vector pulse width modulation (SVPWM) inverter vector voltage has a corresponding stator flux increment, which are shown in Fig. 2 [29], [30]. Hysteresis controllers are implemented for the control of the stator flux magnitude. As a result, the flux can be maintained within the specific hysteresis band through the selection of appropriate stator flux increments. The corresponding trajectory of the stator flux within the hysteresis band is shown in Fig. 3. [29], [30]. The stator flux and electromagnetic torque are controlled by a two- and three-level hysteresis controller respectively [29], [30]. The appropriate switching states of the inverter are determined from a look-up table, in which the outputs of the hysteresis controllers and position of the stator flux are used.

The look-up table implemented as part of the conventional DTC model studied in this paper is shown in Table 5 [29], [30].

**Table 5:** Look-Up Table for CDTC Stator Voltage Vectors [29], [30].

*HT*_*e*_	*H*_*ψ*_	S1	S2	S3	S4	S5	S6
1	1	${\bar{V}}_{2}$	${\bar{V}}_{3}$	${\bar{V}}_{4}$	${\bar{V}}_{5}$	${\bar{V}}_{6}$	${\bar{V}}_{1}$
0	1	${\bar{V}}_{0}$	${\bar{V}}_{7}$	${\bar{V}}_{0}$	${\bar{V}}_{7}$	${\bar{V}}_{0}$	${\bar{V}}_{7}$
-1	1	${\bar{V}}_{6}$	${\bar{V}}_{1}$	${\bar{V}}_{2}$	${\bar{V}}_{3}$	${\bar{V}}_{4}$	${\bar{V}}_{5}$
1	−1	${\bar{V}}_{3}$	${\bar{V}}_{4}$	${\bar{V}}_{5}$	${\bar{V}}_{6}$	${\bar{V}}_{1}$	${\bar{V}}_{2}$
0	−1	${\bar{V}}_{7}$	${\bar{V}}_{0}$	${\bar{V}}_{7}$	${\bar{V}}_{0}$	${\bar{V}}_{7}$	${\bar{V}}_{0}$
-1	−1	${\bar{V}}_{5}$	${\bar{V}}_{6}$	${\bar{V}}_{1}$	${\bar{V}}_{2}$	${\bar{V}}_{3}$	${\bar{V}}_{4}$

The outputs of the hysteresis controllers are given by equations (16) and (17), and are found with the use of the error in the electromagnetic torque and stator flux, indicated by $\Delta T_{e}$ and $\Delta \psi_{s}$ respectively [29], [30]. The hysteresis band widths controlling the electromagnetic torque and stator flux are represented by $HB_{Te}$ and $HB_{\psi }$ respectively.

(16) $$HT_{e}=\left\{ \begin{array}{cc} 1\; & for\;\Delta T_{e}>HB_{Te}\\ 0\; & for\;-HB_{Te}<\Delta T_{e}<HB_{Te}\\ -1\; & for\;\Delta T_{e}<-HB_{Te} \end{array}\right.$$

(17) $$H_{\psi }=\left\{ \begin{array}{cc} 1\; & for\;\Delta \psi_{s}>HB_{\psi }\\ -1\; & for\;\Delta \psi_{s}<-HB_{\psi } \end{array}\right.$$

As DTC mechanisms control the electromagnetic torque directly, a PI speed control loop is required for the desired speed to be obtained. A torque reference corresponding to the desired speed is generated by the PI controller in the speed control loop. Table 6 indicates the parameter values used in various parts of the DTC model studied. The hysteresis band limits were chosen in order to control the values of the stator flux and electromagnetic torque. The stator flux hysteresis controller limits the maximum and minimum values of the stator flux, resulting in a circular stator flux trajectory created by the boundaries of the hysteresis band (seen in Fig. 3) [30]. The stator flux hysteresis controller has very little impact on the torque ripple, which is controlled mainly through the torque hysteresis band. In general, the torque ripple responds proportionally to changes in the limits of the torque hysteresis band; however, lower torque hysteresis band limits result in an increased switching frequency and proportional increase in inverter switching losses [30].

**Table 6:** CDTC Model Parameters.

Parameter	Value
Torque Hysteresis Controller Band Limits (*HB*_*Te*_)	Lower Limit = −1 Upper Limit = 1
Flux Hysteresis Controller Band Limits (*HB*_*ψ*_)	Lower Limit = −0.02 Upper Limit = 0.02

Lastly, adequate operation of the DTC system requires that a certain DC voltage level can be supplied to the inverter, which must also be calculated. Investigation into the maximum modulation index of circular flux trajectory direct torque control is provided by the researchers in [32]. However, a study on the modulation index of SVPWM is also provided in reference [32]. This investigation leads to equation (18), which enables the DC link voltage for an SVPWM switching scheme-based inverter to be determined [32].

(18) $$V_{dc}=\frac{V_{fund}}{m\times \frac{2}{\pi }}$$

Where; $V_{dc}$ represents the required inverter supply voltage, the fundamental component of the phase amplitude generated from the pulse-width-modulated switching sequence is given by $V_{fund}$, and the modulation index of the inverter and corresponding switching scheme is represented by *m*. Circular flux trajectory DTC can also be achieved while operating the inverter in the overmodulation range. Based on the findings in [32], the modulation index and DC link values applicable to the DTC mechanism implemented in this article are shown in Table 7. Notably, a circular flux trajectory can be ensured at a lower voltage when overmodulation is utilized; however, linear modulation was utilized in this study.

**Table 7:** Overmodulation and DC Link Parameters for the CDTC Control Mechanism.

Parameter	Value
Linear range modulation index	0.907
Overmodulation range modulation index	0.950
DC Voltage required for linear modulation	565.62 V
DC Voltage required for overmodulation	540.02 V

## 5 Conventional direct torque results

**Figure 4:**
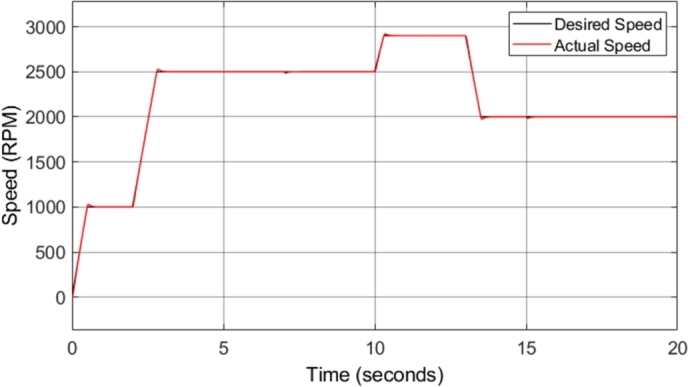
CDTC – Induction motor speed.

A 1 MHz sampling frequency was utilized in this study, as the sampling frequency has a major impact on the ripples observed. Fig. 4 shows the induction motor speed response in comparison to the reference or desired speed. Ramp speed inputs were utilized during the simulation due to the nature of acceleration in vehicle systems. The figure shows that the motor drive system responds well to acceleration and deceleration, with very little noticeable overshoot or undershoot. However, the maximum speed at which the motor was driven was 2900 r.p.m. The rated speed of the induction motor utilized was 2952 r.p.m., and this was not exceeded as field-weakening control was not implemented in the DTC mechanism. As mentioned previously, implementation of field-weakening control is required to operate the motor above its rated speed specification, and is therefore required in order to operate the motor at the maximum required speed of 8046 r.p.m.

**Figure 5:**
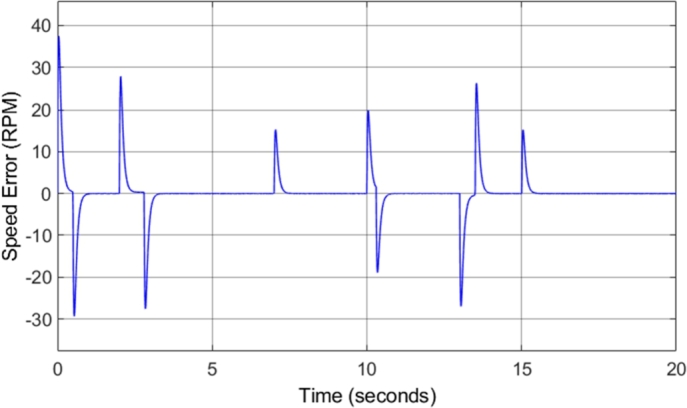
CDTC – Induction motor speed error.

Fig. 5 shows the induction motor speed error when compared to the desired or reference speed, utilizing the drive cycle shown in Fig. 4. The speed error graph indicates that desirable results were achieved as an overshoot/undershoot of less than 3% was obtained in all speed changes in the drive cycle. In addition, there is no steady-state error. The implemented mechanism allows for a fast response, minimal overshoot, and no noticeable steady-state error.

**Figure 6:**
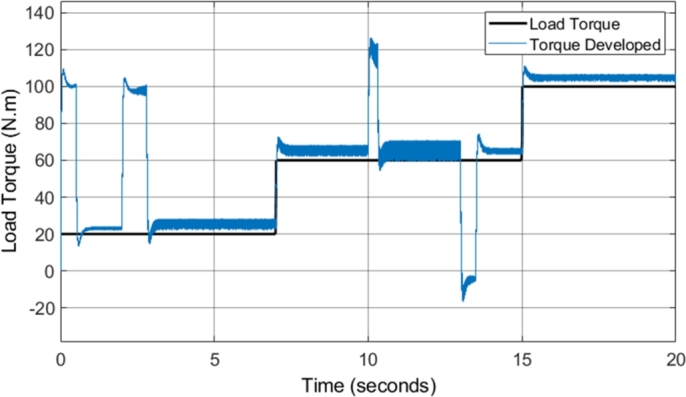
CDTC – Torque developed by Induction motor (1 MHz).

**Figure 7:**
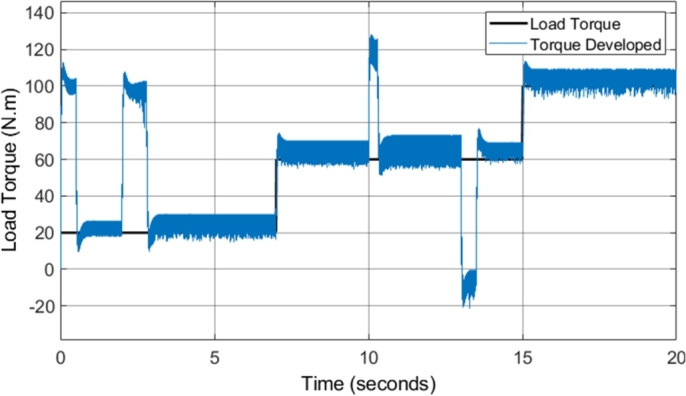
CDTC – Torque developed by Induction motor (200 kHz).

Fig. 6 shows the developed torque that the induction motor supplied to the load in comparison to the load torque. Several notable results can be highlighted from the developed torque. Initially, when accelerating, the motor must supply a greater torque than is required by the load, in order to allow for the desired speed to be achieved. Fig. 6 indicates that this is the case during all periods in which the motor is accelerating. Additionally, when decelerating, the developed torque is lower than the load torque. The DTC model implemented provides fast torque response with very little overshoot, a major benefit in the use of DTC schemes. The torque developed responds well to step inputs in the load torque, with no noticeable delay in the response. However, one of the issues that can be noticed is the large torque ripple, which is especially apparent when the motor is operated at higher speeds. When the motor is operating at 2900 r.p.m., a torque ripple of approximately 11 N-m can be observed. The torque ripple is directly proportional to the width of the torque controller hysteresis band. However, there is a delay between the sampling of the values and the changing of the appropriate switching states in digital implementation. As a result, the torque ripple is dependent on the hysteresis controller as well as the sampling frequency of the digital controller. Reducing the torque controller hysteresis band width too much can increase the torque ripple, as incidents of overshoot in the estimated torque, that are above the hysteresis band, can cause the reverse voltage vector to be selected resulting in sharp decreases in torque. The torque graph shown in Fig. 6 was generated with a simulation sampling frequency of 1 MHz. Fig. 7 shows the torque developed by the induction motor with a sampling frequency of 200 kHz. It can be observed that the developed torque ripple is noticeably higher than when a sampling frequency of 1 MHz was used. The result indicates the strong dependence of the DTC mechanism on the processor speed.

**Figure 8:**
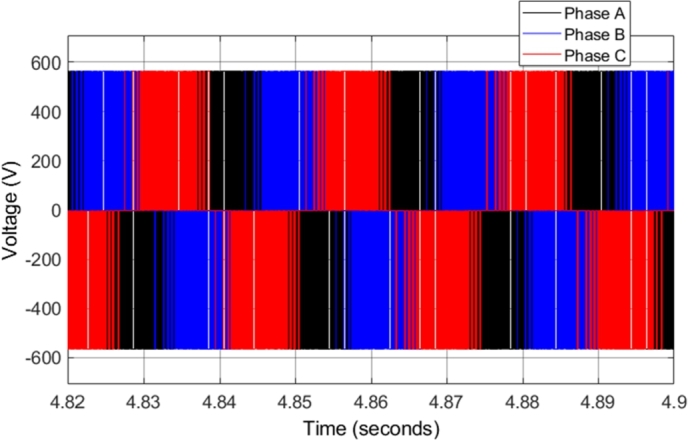
CDTC – Three-phase inverter voltage.

Fig. 8 shows the steady-state voltage generated from the two-level three-phase inverter supplying the induction motor when the motor is operating at 2500 r.p.m. All three-phases are shown, indicating the phase relationship of the voltages. One of the issues found in CDTC is a variable inverter switching frequency, as the torque and flux slopes vary with operating conditions, which affects the switching controlled by the hysteresis controllers.

**Figure 9:**
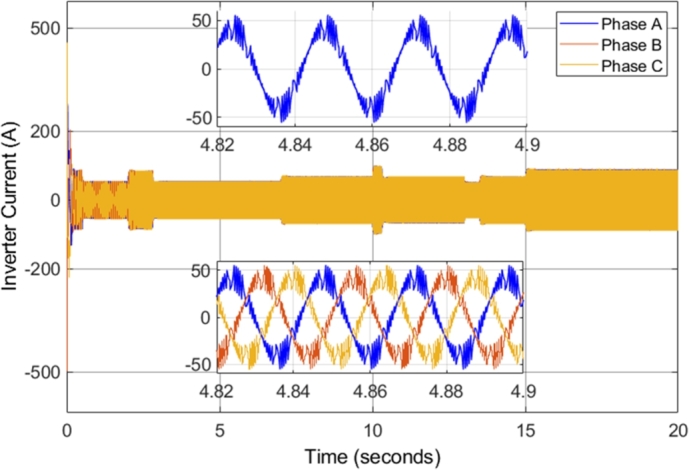
CDTC – Three-phase inverter current.

Fig. 9 shows the inverter current supplied to the induction motor during the entire drive cycle. In addition, zoomed-in portions of both the Phase A and the three-phase steady-state inverter current supplied to the induction motor, when the motor is operating at 2500 r.p.m. with a load torque of 20 N-m, are shown. The inverter current is sinusoidal; however, there is a high current ripple (harmonic content). The high current ripple is one of the disadvantages of conventional direct torque control, as mentioned in the theoretical review previously. The three-phase inverter current shown presents an expected result, as the currents in each of the three phases are separated by 120 degrees, as expected in a three-phase system. Notably, the current has a peak amplitude of approximately 50 A when the load torque is 20 N-m. However, Fig. 9 shows the change in the current when the load torque which the motor is supplying is increased. From 15 to 20 seconds, the induction motor is operating at 2000 r.p.m. with a load torque of 100 N-m. The result clearly indicates the relationship between the current drawn by the motor and the load torque.

**Figure 10:**
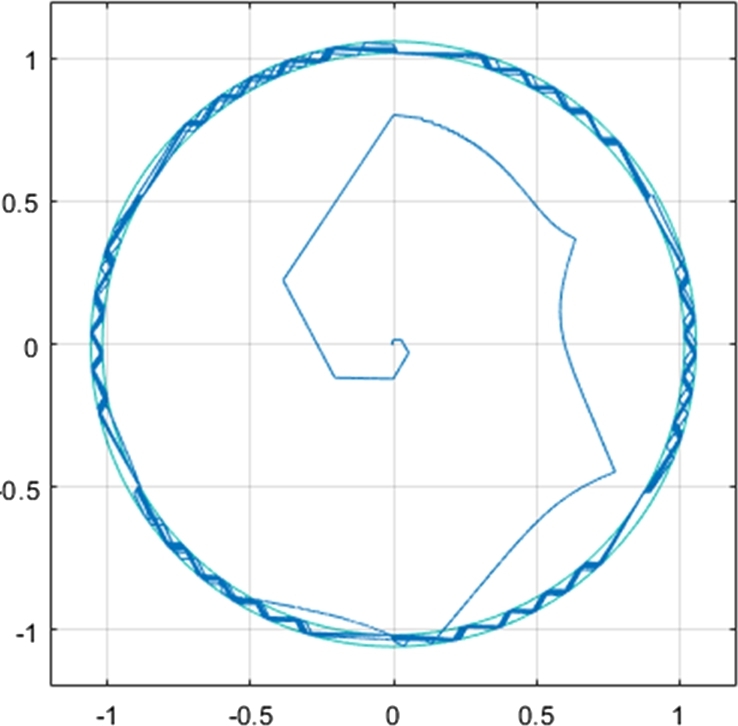
CDTC – Stator Flux Trajectory.

Fig. 12 shows the q- and d-axis components of the stator flux. Notably, the flux in both the q- and d-axes have an amplitude of approximately 1.04 Wb, as controlled by the stator flux magnitude reference. The reference stator flux magnitude was kept constant at 1.04 Wb throughout the simulation, and as a result, Fig. 12 shows an expected result. Although Fig. 12 confirms that the stator flux in the q- and d-axes have the correct magnitude, a more notable result can be seen by plotting the flux values on the d-q axis. The plot results in the stator flux trajectory being generated, which is shown in Fig. 10.

**Figure 11:**
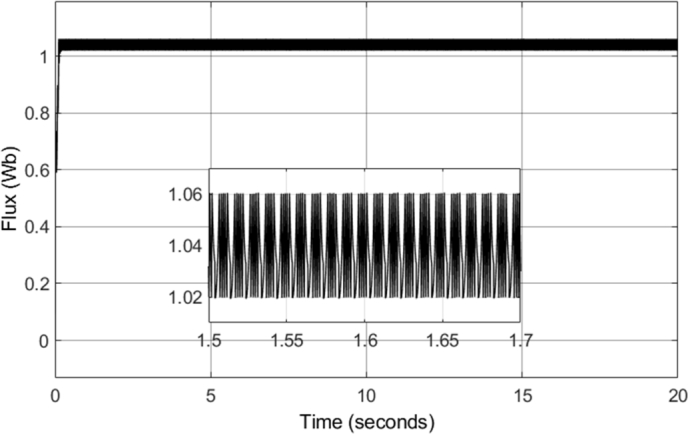
CDTC – Stator Flux Magnitude.

The stator flux trajectory shown is equivalent to the theoretical stator flux trajectory of conventional DTC, shown in Fig. 3. The result shows that the flux initially starts outside of the hysteresis band circles, which occurs at start-up. However, the flux vector, after a few switching sequences, follows the circular flux trajectory. The circles plotted have radii of 1.02 and 1.06, indicating the limits of the flux hysteresis band controllers. The result achieved is a desirable one, as the stator flux (excluding the initial switching sequences) follows a circular flux trajectory, and remains inside of the hysteresis band. Such a result indicates that the correct DC voltage has been supplied to the inverter, in order to enable circular flux trajectory DTC. In addition, it also indicates that the flux hysteresis controller is operating expectedly. The stator flux is forced between the hysteresis band, as the stator flux increments are being correctly selected. This can be further confirmed by the stator flux magnitude plot shown in Fig. 11. The flux magnitude remains constant throughout the 20-second drive cycle, with the ripple maintained between 1.02 and 1.06 Wb. This can be seen more clearly in the zoomed-in portion of the plot, indicating the ripple when the motor is operating in steady-state conditions, with a speed of 2500 r.p.m. and a load torque of 20 N-m.

**Figure 12:**
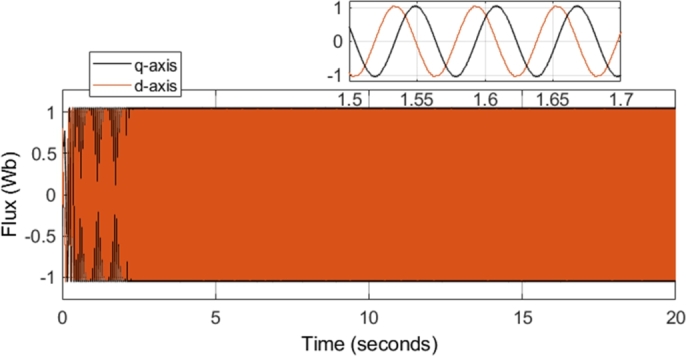
CDTC – Stator Flux (d- and q- axis).

The DTC control mechanism implemented produces expected results for conventional DTC, implemented with the use of hysteresis controllers. The speed response obtained is a very favorable result, as the reference speed is tracked with very little overshoot and no steady-state error. In addition, the developed torque of the motor responds quickly to changes in the load torque with minimal overshoot. The stator flux trajectory observed perfectly matches the theoretical result expected. The stator flux vector rotates in a circular manner, contained within the hysteresis band controller limits. However, despite some of the favorable results obtained, issues such as high torque ripple, high current ripple (harmonic distortion), and variable switching frequency were observed. In addition, it was observed that a high-speed processor is required for adequate implementation of conventional DTC. The issues observed are undesirable for implementation in EV applications. The authors in [30], [31] propose methods in which these issues can be mitigated. The methods include the implementation of different switching control strategies, different voltage SVM techniques, predictive control schemes, and intelligent or fuzzy logic control schemes [30], [31].

## 6 DTC-SVM – improvements to CDTC

The results obtained from the simulation of conventional DTC highlighted the disadvantages associated with the use of hysteresis controllers in the system, as well as the issues created from a variable switching frequency. Such disadvantages, including high electromagnetic torque, stator flux and current ripples, as well as current distortions are a common trend in literature and are documented by the researchers in [28], [29], [31]. As a result, extensive research has been conducted surrounding the improvement of conventional DTC. The characteristics of the improved methods developed in comparison to conventional DTC are shown in Table 8. The methods reviewed in Table 8 are Space Vector Modulation DTC (DTC-SVM), model predictive DTC (DTC-MPC), fuzzy logic-based DTC (fuzzy DTC), artificial neural network-based DTC (ANN DTC) and sliding mode-based DTC (SMC DTC).

**Table 8:** Comparison of Improvement Techniques for DTC Systems [31], [33], [35].

Metric	CDTC	DTC-SVM	DTC-MPC	Fuzzy DTC	ANN DTC	SMC DTC
Dynamic torque response	Fast	Fast	Fast	Very Fast	Very Fast	Fast
Torque & flux ripple	High	Low	Low	Very Low	Very Low	Medium
Current THD	High	Lower	Lower	Lower	Lower	Lower
Switching Frequency	Variable	Constant	Constant	Constant	Constant	Almost Constant
Computation time	Low	Medium	Medium	High	High	High
Switching Loss	High	Low	Low	Low	Low	Medium
Algorithm Complexity	Simple	Simple	Simple	More Complex	More Complex	Complex

As the DTC-SVM technique enables improved performance (reduced ripples, reduced current distortion, and constant switching frequency) with simple algorithm complexity, it is chosen for investigation in this paper. The objective is to mitigate the issues observed in the conventional DTC system investigated. However, there are various other suitable control methods given in Table 8 which could also have been chosen. SMC DTC was avoided as it has higher switching losses, a larger required computation time, and a more complex algorithm construction. In addition, sliding mode control can cause undesired chattering in the quantity being controlled as a result of the discontinuous section of the control mechanism [2]. Table 8 indicates that DTC-MPC has very similar performance characteristics to the DTC-SVM technique chosen in this article. However, DTC-MPC requires both the use of a predictive model (to predict the future behaviour of the system), as well as a pre-defined cost function, which allows for optimization of the control outputs [2]. Furthermore, the authors in [33] suggest that hysteresis regulation is used in conventional DTC-MPC. In comparison, DTC-SVM makes use of a PI controller with fundamental motor and DTC calculations. A method not evaluated in Table 8 is deadbeat control based DTC schemes. Deadbeat control utilizes different control strategies in steady state and transient operation; however, the researchers in [34] suggest that deadbeat control is sometimes not feasible due to the limitation of inverter voltages and currents.

**Figure 13:**
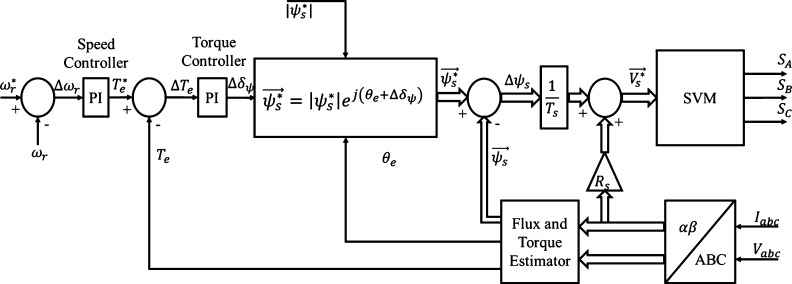
DTC-SVM system with closed-loop torque control (This figure was replicated from [2] - DOI: 10.1109/ACCESS.2021.3110736, using concepts found in [32], [33], [34]).

DTC-SVM techniques correctly select switching states for the voltage source inverter with the implementation of a voltage modulator [36]. As result, the hysteresis controllers and switching table utilized in CDTC systems are no longer required in DTC-SVM. However, the hardware topology present in DTC-SVM is still similar to that utilized in conventional DTC [35]. As with conventional DTC, only the stator parameters of the IM are required for adequate operation of the DTC-SVM system. The implemented SVM technique aims to allow for reduced torque/flux ripples and harmonic distortion in the current waveform through optimal selection of the inverter switching states and maintaining a constant switching frequency. Three different variations of the DTC-SVM control structure exist, which are DTC-SVM using closed-loop flux control, DTC-SVM using closed-loop torque control, and DTC-SVM using both closed-loop torque and flux control [35]. The DTC-SVM closed-loop torque control structure was chosen for implementation in this paper as it reduces the flux, torque, and current ripples, with a simple control structure. The control structure can be implemented solely in the stationary reference frame and utilizes the changes in the developed torque to appropriately select the required voltage vector [36]. DTC-SVM with closed loop torque control is largely based on the control of the load angle, addressing the limitations observed in CDTC [35], [36]. The load angle reference criteria are met through the selection of the optimal voltage vector, appropriately changing the stator flux [35]. The proposed control structure to be implemented in this paper is shown in Fig. 13.

The DTC-SVM system proposed utilizes the same flux and torque estimator model discussed in conventional DTC, and as a result, the equations are not redefined in this section. Conventional DTC enables the adjustment of the developed electromagnetic torque, through the control of the angle between the stator and rotor magnetic flux vectors ($\theta_{e}$ as previously defined) [35], [37]. The same concept is of particular importance in the DTC-SVM control structure proposed. As can be seen in Fig. 13, the output of the torque PI controller is $\Delta \delta_{\psi }$, which represents a desirable change in angle between the stator and rotor flux vectors, based on the electromagnetic torque error [35], [37]. The desired change in angle is added to the estimated angle of the stator flux vector, and as a result, the stator flux reference can be estimated using equation (19) [35], [37].

(19) $$\psi_{s}^{⁎}=\left|\psi_{s}^{⁎}\right|e^{j\left(\theta_{e}+\Delta \delta_{\psi }\right)}$$

The desirable change in stator flux can be calculated by subtracting the instantaneous stator flux from the reference flux vector. As a result, the optimal stator voltage vector can be determined using equation (20), allowing for the voltage source inverter to be driven through the SV-PWM technique [35], [37].

(20) $$\overset{\to }{V_{s}^{⁎}}=\frac{\Delta \overset{\to }{\psi_{s}}}{T_{s}}+R_{s}\overset{\to }{I_{s}}$$

DTC-SVM techniques allow for change in the stator flux in any direction, producing smoother stator flux oscillations [37]. The conventional DTC structure can be easily adapted in order to incorporate the changes and improvements of DTC-SVM with closed loop torque control. The objective of the implementation of the DTC-SVM system is to enable improved performance, ensuring the control mechanism is more suitable for EV applications.

## 7 DTC-SVM results

A 1 MHz sampling frequency was utilized in the study of the DTC-SVM model, as the sampling frequency has a major impact on the ripples observed. In addition, an SVM switching frequency of 20 kHz was utilized.

**Figure 14:**
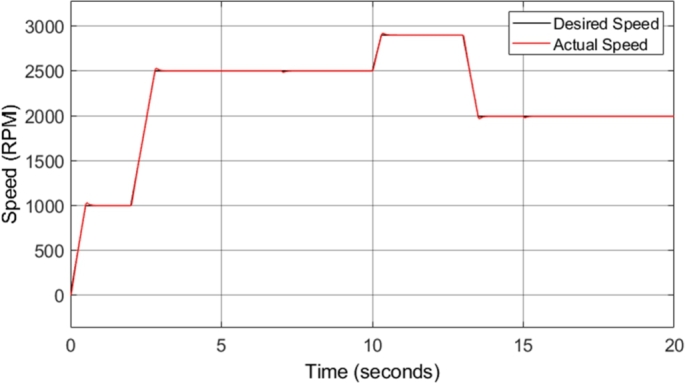
DTC SVM – Induction motor speed.

**Figure 15:**
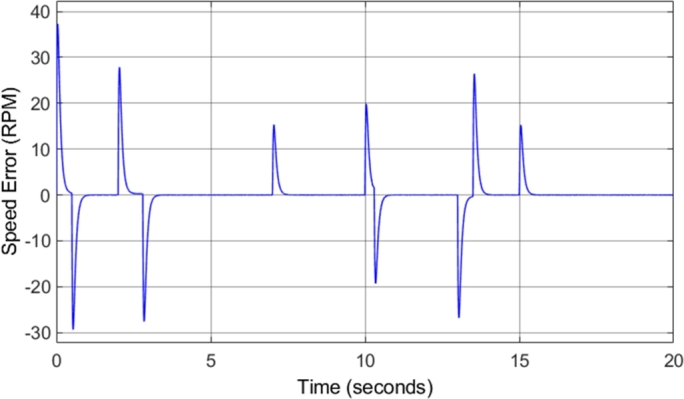
DTC SVM – Induction motor speed error.

Fig. 14 shows the induction motor speed response in comparison to the reference or desired speed. The drive cycle used to test the conventional DTC system was utilized again for the DTC-SVM system in order to offer comparable results. The figure shows that the motor drive system responds well to acceleration and deceleration, with very little noticeable overshoot or undershoot. Fig. 15 shows the induction motor speed error when compared to the desired or reference speed. The speed error graph indicates that desirable results were achieved as an overshoot/undershoot of less than 3% was obtained in all speed changes in the drive cycle. In addition, there is no steady-state error. The result obtained is almost identical to the results obtained from the conventional DTC system, indicating that the DTC-SVM mechanism chosen has no undesirable effects on the speed response of the system.

**Figure 16:**
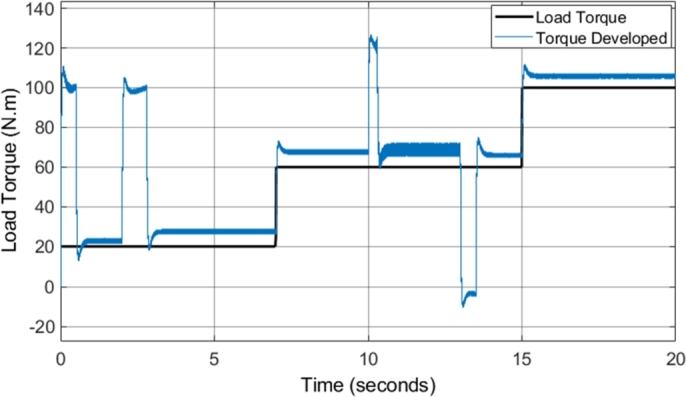
DTC SVM – Torque developed by Induction motor (1 MHz).

Fig. 16 shows the developed torque that the induction motor supplied to the load in comparison to the load torque. The result highlights several notable improvements when compared to the torque developed by the CDTC system. The DTC-SVM model implemented provides fast torque response with very little overshoot, a major benefit in the use of DTC schemes. The torque developed responds well to step inputs in the load torque, with no noticeable delay in the response. However, the replacement of the hysteresis controllers and the switching table in CDTC allows for the significant reduction in the torque ripple in most operating conditions. The reduction in torque ripple is illustrated in Table 9. An average overall reduction of approximately 26% is observed, showing that the implementation of the DTC-SVM scheme significantly assists in the reduction of the unwanted torque ripple in CDTC. However, one of the issues that can be noticed with the DTC-SWM scheme implemented is the presence of steady-state torque error. The steady-state torque error is higher with operation at higher speeds, and is a result of the torque being controlled from the speed error. The speed PI controller provides a torque reference which is required to maintain the desired speed, and as a result, in certain operating conditions a higher torque is required. This is a characteristic of the DTC mechanism implemented, and is also noted in a similar manner by the authors in [38].

**Table 9:** Torque Ripple – CDTC vs DTC-SVM.

Condition	CDTC Ripple (N-m)	DTC-SVM Ripple (N-m)	% Change
1000 rpm 20 N-m	2	3	+50.00
2500 rpm 20 N-m	6	2.65	−55.83
2500 rpm 60 N-m	6.1	2.7	−55.74
2900 rpm 60 N-m	11.2	7.05	−37.05
2000 rpm 60 N-m	3.8	2.7	−28.95
2000 rpm 100 N-m	4	2.85	−28.75
**Average**			−**26.05**

**Figure 17:**
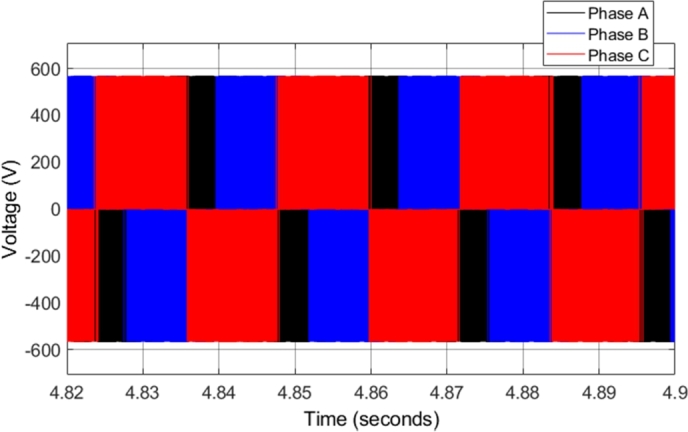
DTC SVM – Three-phase inverter voltage.

Fig. 17 shows the steady-state voltage generated from the two-level three-phase inverter supplying the induction motor when the motor is operating at 2500 r.p.m. Fig. 17 shows all three-phases, indicating the phase relationship of the voltages. The only significant difference between the voltage waveforms in CDTC and DTC-SVM is that the voltage waveforms in the DTC-SVM scheme are generated with a constant switching frequency.

**Figure 18:**
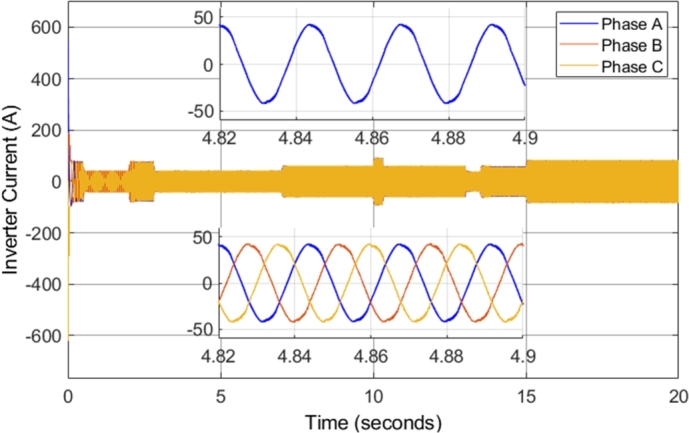
DTC SVM – Three-phase inverter current.

Fig. 18 shows the inverter current supplied to the induction motor during the entire drive cycle. In addition, zoomed-in portions of both the Phase A and the three-phase steady-state inverter current supplied to the induction motor, when the motor is operating at 2500 r.p.m. with a load torque of 20 N-m, are shown. The inverter current is sinusoidal, and the current magnitude is proportional to the torque developed by the motor. The currents in each of the three phases are separated by 120 degrees, as expected in a three-phase system. However, the current in the DTC-SVM system has significantly reduced current ripple and distortion. In steady-state, when the motor is operating at 2500 r.p.m. with a load torque of 20 N-m, the current ripple is decreased by approximately 90% when compared to CDTC. This is a significant result, as large current ripple is a major disadvantage of conventional DTC, which DTC-SVM is able to mitigate. As a result, the peak current is decreased, and the quality of the power supplied to the motor is improved.

**Figure 19:**
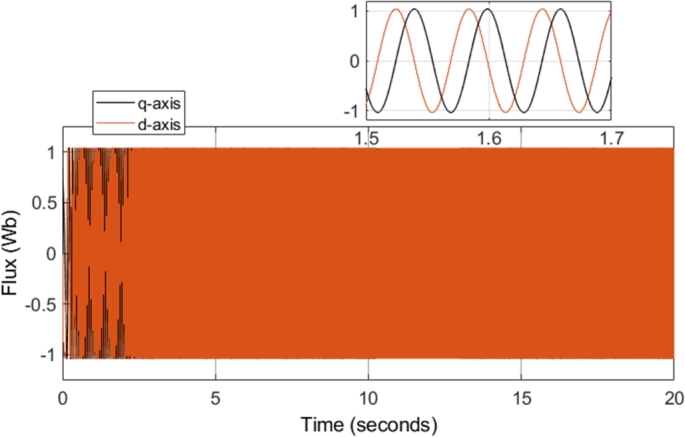
DTC SVM – Stator Flux (d- and q-axis).

**Figure 20:**
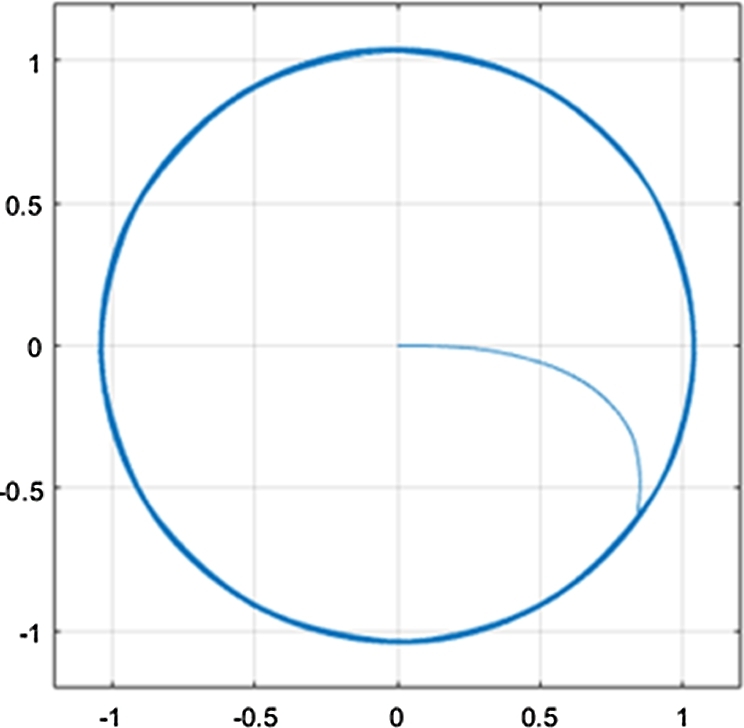
DTC SVM – Stator flux trajectory.

**Figure 21:**
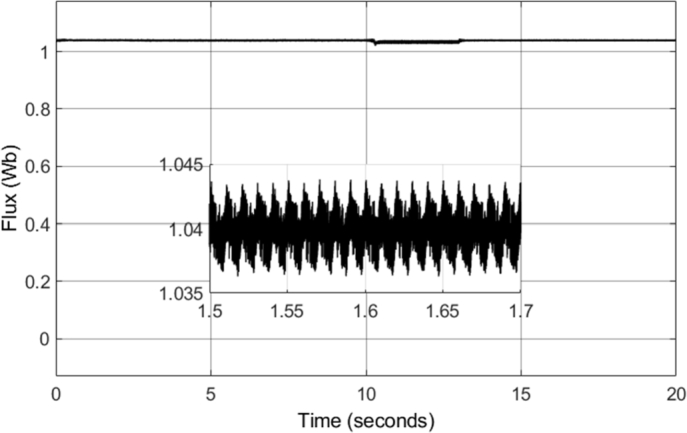
DTC SVM – Stator Flux Magnitude.

Fig. 19 shows the q- and d-axis components of the stator flux. Notably, the flux in both the q- and d-axes have an amplitude of approximately 1.04 Wb, as controlled by the stator flux magnitude reference. A constant magnitude of 1.04 Wb was used for the stator flux reference throughout the simulation, and therefore, Fig. 19 shows expected results. However, more notable results can be seen in Fig. 20 and Fig. 21. Fig. 20 shows that a smooth circular flux trajectory is generated when the induction motor is controlled using the DTC-SVM structure. The result obtained is improved when compared to CDTC, as the circular flux band width is narrower. The zoomed-in portion of the plot in Fig. 21 indicates the ripple when the motor is operating in steady-state conditions, with a speed of 2500 r.p.m. and a load torque of 20 N-m. As can be seen, a ripple of approximately 8.2 mWb can be observed, which is approximately an 80% reduction in the flux ripple observed in CDTC. The ripple increases slightly at 2900 r.p.m.; however, it is still reduced when compared to the ripple observed in CDTC.

The implementation of DTC-SVM using closed-loop torque control presents significant advantages when compared to the CDTC system investigated. Large reductions in torque, flux, and current ripple (of 26.05%, 80%, and 90% respectively) are noticed, with the method also enabling a constant switching frequency. As a result, the DTC-SVM technique presented significantly improves the CDTC structure initially simulated and is more applicable for implementation in EV systems. The DTC-SVM system can therefore be further investigated for use in EV systems through the integration of field-weakening and sensorless control. Despite the favourable results obtained, further investigation into the improvement of the DTC mechanism can be made. Such investigation may include the implementation of DTC-SVM with both closed-loop torque and flux control as presented in [39], or investigation into other improvement techniques proposed by the authors in [30], [31]. Such further investigation would aim to further reduce ripple and mitigate steady-state torque error. However, a comprehensive investigation of DTC-SVM with closed-loop torque control is provided in this paper.

## 8 Field-weakening control

**Figure 22:**
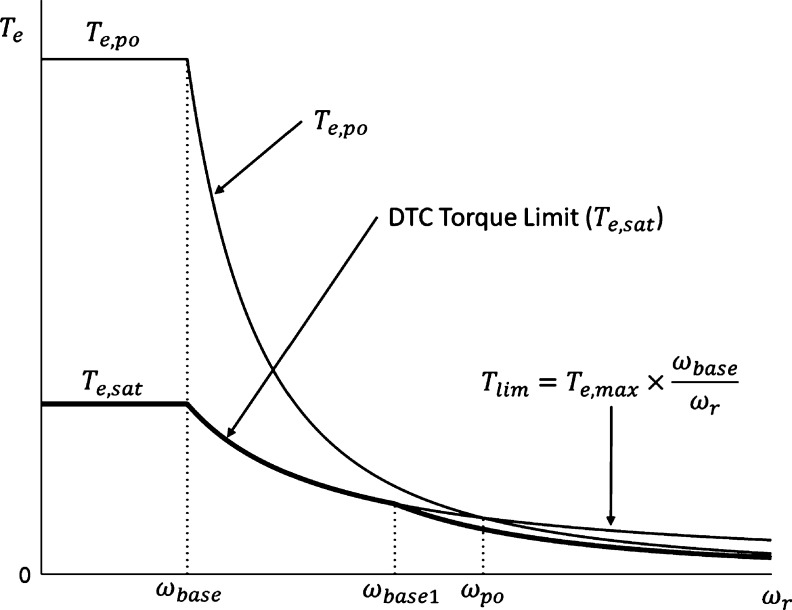
Torque limiting in the field-weakening region (This figure was replicated from [2] - DOI: 10.1109/ACCESS.2021.3110736, using concepts found in [15]).

High-speed operation of the traction motor is frequently required due to the application nature of EVs and the speed requirements of highway driving. However, high-speed operation (above the rated speed specification) of an induction motor, when utilizing vector control mechanisms, is limited by the maximum current rating of the motor windings as well as the maximum inverter voltage [2], [40]. As a result, the integration of field-weakening control is required to allow the traction motor to operate above the rated speed specification [40], [41]. The inverter current rating and DC link voltage limit the motor torque in the field-weakening region, and as a result, motor torque limits are required to ensure stable operation of the induction motor in the field-weakening region. The authors in [41] provide research in this area, with the proposal of a field-weakening method which allows for good dynamic performance and stable operation of the motor across the entire speed range of the drive. Such drive performance is ensured with the implementation of reference torque limits. The method proposed was chosen for use in this study due to its suitability for use in direct torque controlled EV drive systems. Operation of a traction motor drive across its entire speed range requires three regions to be considered, which are depicted in Fig. 22. The pull-out torque of the traction motor, as well as the maximum machine overload torque (which is extended to the field-weakening region) define the torque limits shown in Fig. 22 [41]. Computation of the stator flux reference value which provides almost optimal stator flux orientation, is carried out with the use of the (1/$\omega_{r}$) field-weakening method [41], [42]. Due to the implementation of this strategy, the stator flux reference in the DTC algorithm is calculated utilizing equation (21) [41].

(21) $$\psi_{s}^{⁎}=\frac{\psi_{s,rated}\omega_{base}}{\left|\omega_{r}\right|}\;for\;\left|\omega_{r}\right|>\omega_{base}$$

In which, $\psi_{s,rated}$ is the rated stator flux, $\omega_{base}$ is the base speed of the drive, which can be calculated using equation (22), and $\omega_{r}$ is the speed of the rotor [41].

(22) $$\omega_{base}=\frac{V_{s,\;max}}{\psi_{s,\;rated}}$$

Where, $V_{s,max}$ is the maximum phase voltage that can be supplied by the inverter. The maximum phase voltage which the inverter can supply is determined by the DC link voltage, the PWM switching strategy, dead-time effects of the inverter, and the ON-state voltage drops of the inverter [41]. The torque limits necessary for stable operation of the traction motor drive, which correspond to Fig. 22, can be calculated utilizing equation (23) [41].

(23) $$\left\{ \begin{array}{c} T_{e,sat}=T_{max},\;if\;\left|\omega_{r}\right|\leq \left|\omega_{base}\right|\\ T_{e,sat}=\frac{T_{max}\omega_{base}}{\left|\omega_{r}\right|},\;if\;\omega_{base}<\left|\omega_{r}\right|\leq \omega_{base1}\\ T_{e,sat}=3\frac{P}{2}\left(\frac{1-\sigma }{L_{s}}\right)\left(\frac{\tau_{r}\omega_{slip1}\psi_{s}^{2}}{1+{\left(\sigma \omega_{slip1}\tau_{r}\right)}^{2}}\right),\\ if\;\left|\omega_{r}\right|>\omega_{base1} \end{array}\right.$$

In which, $T_{e,sat}$ is the torque saturation limit required in each region of operation, $T_{max}$ is the maximum machine overload torque, $\omega_{base1}$ is a speed which is based on $\omega_{po}$ (the speed at which the maximum machine overload torque and the pull-out torque intersect in the field-weakening region, as shown in Fig. 22), *P* is the number of pole pairs in the induction motor, $\omega_{slip1}$ is the slip frequency which corresponds to $\omega_{base1}$, *σ* is the total leakage factor ($\sigma =1-L_{m}^{2}/L_{s}L_{r}$), and $\tau_{r}$ is the rotor time constant ($\tau_{r}=L_{r}/R_{r}$). $L_{s}$ and $L_{r}$ are the stator and rotor self-inductances respectively, and $L_{m}$ is the magnetising inductance of the induction motor. As a result, for the torque limits to be calculated, various other terms need to be calculated and defined. The speed $\omega_{po}$ can be calculated using equation (24) [41].

(24) $$\omega_{po}=3\frac{P}{2}\left(\frac{1-\sigma }{2\sigma L_{s}}\right)\left(\frac{\psi_{s,rated}^{2}}{T_{max}}\right)\omega_{base}$$

A practically employed field-weakening technique should use a boundary speed ($\omega_{base1}$), which is slightly less than $\omega_{po}$. This suggests that the boundary speed utilized should be slightly less than the theoretical boundary speed calculated, limiting the motor torque to slightly less than the pull-out torque of the machine [41]. As a result, for the purpose of this investigation, the boundary speed ($\omega_{base1}$) was chosen to be 95% of the theoretical boundary speed ($\omega_{po}$). The slip frequency, which corresponds to $\omega_{base1}$ can be calculated using equation (25) [41].

(25) $$\omega_{slip1}=\frac{1-\sqrt{1-{\left(2K\sigma \right)}^{2}}}{2K\sigma^{2}\tau_{r}}$$

In which *K* can be given by equation (26).

(26) $$K=\frac{T_{max}L_{s}}{3\frac{P}{2}\left(1-\sigma \right)\left(\frac{\omega_{base}}{\omega_{base1}}\right)\psi_{s,rated}^{2}}$$

**Figure 23:**
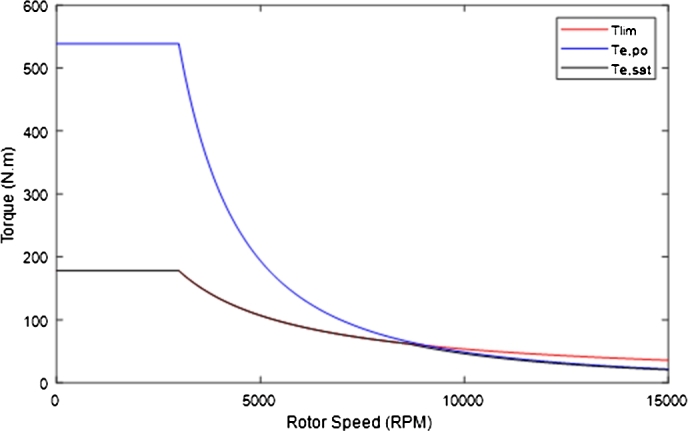
Calculated Torque limits for field-weakening operation.

The torque limits in the field-weakening region are determined through on-line calculation and are dependent on the operating conditions of the motor. However, the speed boundaries of each section of the field-weakening region must be designed using Equations (22) and (24). The calculated speed boundaries required for field-weakening in this study are shown in Table 10. Fig. 23 shows the motor torque limits, and the torque saturation limit calculated in order to maintain stable operation of the traction motor throughout the speed range. The torque saturation limits implemented were 90% of the calculated saturation limits, in order to account for torque ripple and ensure the motor maintains stable operation.

**Table 10:** Speed Limits in the Field-Weakening Region.

Parameter	Value	Parameter	Value
*ω*_*base*_	3000 r.p.m.	*ω*_*base*1_	8624 r.p.m.
*ω*_*po*_	9075 r.p.m.

## 9 Field-weakening control results

**Figure 24:**
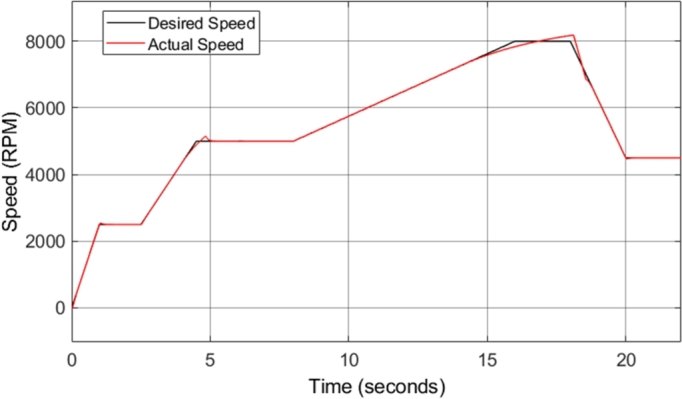
DTC SVM with FW – Induction motor speed.

The fundamental structure of the DTC-SVM mechanism is unchanged when field-weakening is added; however, a dynamic flux reference calculator, as well as the torque limits discussed in the previous section are also incorporated into the model. Fig. 24 shows the motor speed in comparison to the reference or desired speed. The initial drive cycle used to test the CDTC and DTC-SVM systems is not applicable to test the field-weakening functionality of the drive system, as field-weakening enables operation of the motor above its base speed. As a result, the drive cycle is adapted to have a maximum speed of 8000 r.p.m., which is approximately the maximum motor speed required.

**Figure 25:**
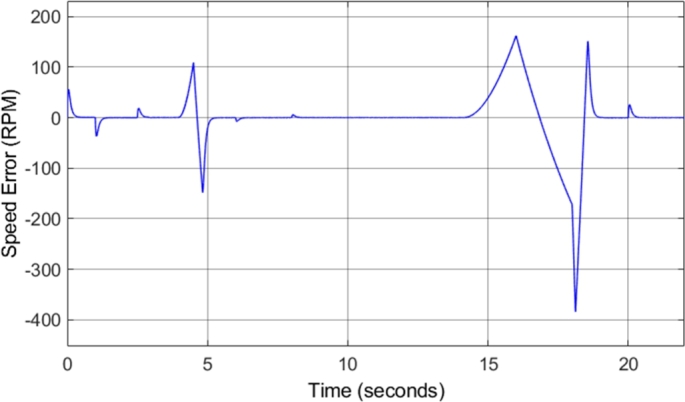
DTC SVM with FW – Induction motor speed error.

Fig. 24 shows that the speed is correctly tracked for most of the drive cycle. However, the result shows that there are sections during which the motor acceleration does not match the desired acceleration, and as a result, the motor takes longer to reach the desired steady-state speed. This is as a result of the torque limiting that is implemented with the incorporation of field-weakening control. The torque is limited to ensure that the motor remains in stable operation. Fig. 25 shows the induction motor speed error when compared to the desired or reference speed. The error graph clearly indicates the sections of the drive cycle in which the desired acceleration is not achieved.

**Figure 26:**
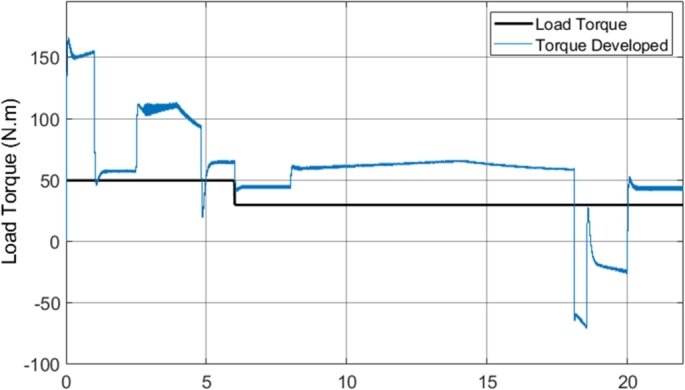
DTC SVM with FW - Torque developed by Induction motor (1 MHz).

Fig. 26 shows the developed torque that the induction motor supplied to the load in comparison to the load torque, when field-weakening was implemented in the DTC-SVM model. The figure indicates that the DTC-SVM scheme produces suitable torque ripple across the entire speed range required (with a maximum speed of more than two times the base speed). A very notable result occurs when the motor is not able to match the desired acceleration (4 to 5 seconds and 14 to 18 seconds). The torque graph shows that the torque is limited in order to maintain stable operation of the motor. The torque limiting is inversely proportional to the speed, governed by the second region in equation (23), as the operating condition of the motor matches the mathematical inequality given by $\omega_{base}<\left|\omega_{r}\right|\leq \omega_{base1}$. The implemented field-weakening algorithm enables the motor to meet the maximum speed requirement, while being forced to accelerate in a stable manner.

**Figure 27:**
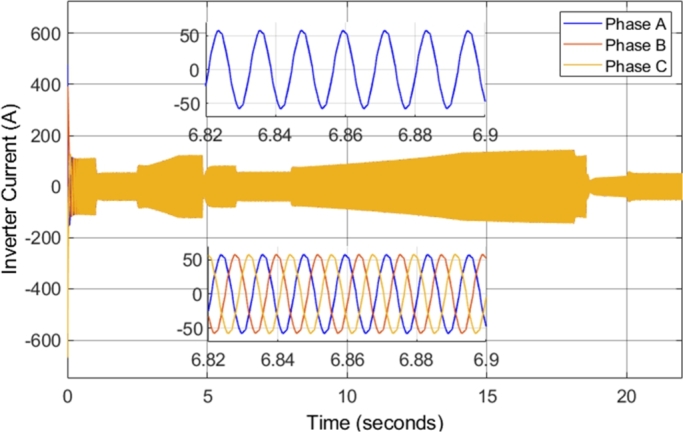
DTC SVM with FW – Three-phase inverter current.

The inverter voltages observed in the simulation remained unchanged as compared to those observed in the DTC-SVM mechanism when field-weakening control had not been implemented. Fig. 27 shows the inverter current supplied to the induction motor during the entire drive cycle. In addition, zoomed-in portions of both the Phase A and the three-phase steady-state inverter current supplied to the induction motor, when the motor is operating at 5000 r.p.m. with a load torque of 30 N-m, are shown. The inverter current is sinusoidal, and the current magnitude is proportional to the torque developed by the motor. The currents in each of the three phases are separated by 120 degrees, as expected in a three-phase system. Sinusoidal current with low distortion or ripple is observed, even in cases when the motor is operated well above the specified base speed. As a result, it can be concluded that the field-weakening control incorporated does not have adverse effects on the inverter current supplied to the motor.

**Figure 28:**
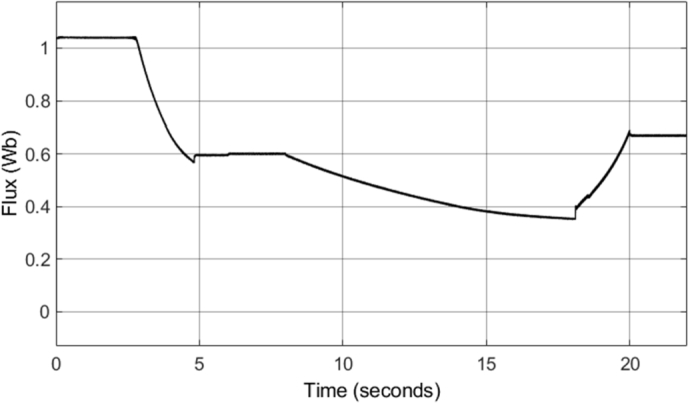
DTC SWM with FW – Stator flux magnitude.

**Figure 29:**
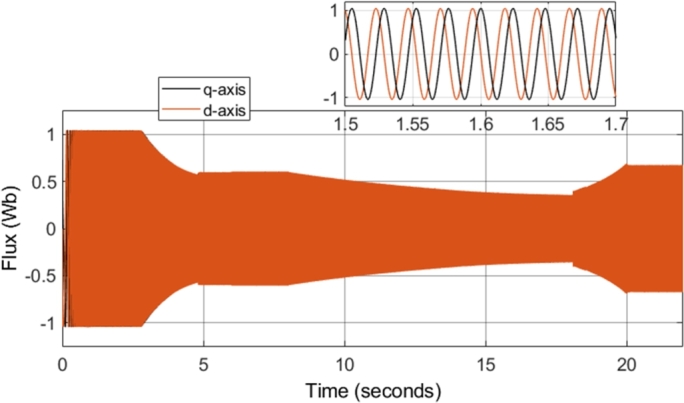
DTC SVM with FW – Stator Flux (d- and q-axis).

The field-weakening method implemented adjusts the stator flux in a manner which is inversely proportional to the rotor speed. However, adjustment of the flux only takes place in operating conditions in which the rotor speed is above the specified base speed. As a result, when the field-weakening control is in operation, the stator flux should be adjusted in a hyperbolic manner. Such adjustment of the stator flux can be seen in the stator flux magnitude graph, shown in Fig. 28. As can be seen, the flux is decreased using field-weakening control between 2.5 to 5 seconds, as well as between 8 to 18 seconds. Furthermore, in periods in which the speed is below the base speed or the speed of the motor is constant, the stator flux magnitude remains constant. This can be further confirmed through the d- and q- axis components of the stator flux, shown in Fig. 29. Additionally, the zoomed-in portion of the stator flux shows smooth sinusoidal oscillations of both the d- and q- axis components.

The field-weakening technique implemented produced favorable results, allowing the traction motor to correctly operate over the entire speed range required. Additionally, the torque is correctly limited, in order to ensure that the motor maintains stable operation throughout the entire drive cycle tested. The results achieved are notable, as field-weakening is essential in traction motor drives utilized in EVs. The results obtained show that a DTC-SVM mechanism, with the implementation of field-weakening control, presents favorable results when applied in electric vehicle systems. The complete mechanism exhibits low torque, current and flux ripples, while achieving fast dynamic response and stable acceleration throughout the speed range required.

## 10 Implementation of sensorless speed control

As an EV system could be considered as a hostile environment, sensorless DTC offers various advantages, which include a reduction in hardware complexity, cost, maintenance, and size of the machine drive, while also allowing for improved noise immunity, and increased reliability [43]. Numerous well-developed induction motor speed estimation methods which can be used within a DTC system are available. These methods include open-loop estimators (which use monitored stator voltages and currents), artificial intelligence-based estimators (neural network, and fuzzy-logic-based systems) and model reference adaptive systems (MRAS) [44], [45]. However, an open-loop rotor flux-based estimation technique was chosen for use in this investigation, as the parameters of the induction motor are completely known, and the method allows for the instantaneous rotor speed to be estimated with the use of basic induction motor model equations [44], [45]. In all of the previous DTC models investigated and implemented in this paper, only the stator parameters were required; however, for on-line estimation of the rotor speed using the rotor flux-based estimation technique, the rotor voltages, currents, and fluxes are also required. The rotor currents in the stationary d- and q-axis can be calculated using equations (27) and (28) respectively [46].

(27) $$i_{dr}=\frac{1}{L_{m}}\left\{\int \left(v_{ds}-R_{s}i_{ds}\right)dt-L_{s}i_{ds}\right\}$$

(28) $$i_{qr}=\frac{1}{L_{m}}\left\{\int \left(v_{qs}-R_{s}i_{qs}\right)dt-L_{s}i_{qs}\right\}$$

The d- and q- axis components of the rotor flux can be found from the rotor current components. The rotor flux in the d- and q-axes are given by equations (29) and (30) respectively [45], [46].

(29) $$\psi_{dr}=L_{r}i_{dr}+L_{m}i_{ds}$$

(30) $$\psi_{qr}=L_{r}i_{qr}+L_{m}i_{qs}$$

As a result, the rotor flux magnitude can be found using equation (31) [46].

(31) $$\left|\psi_{r}\right|=\sqrt{\psi_{dr}^{2}+\psi_{qr}^{2}}$$

Accurate estimation of the flux-linkage components is essential, as it directly impacts the accuracy of the speed estimation system [44]. The estimation technique chosen has been utilized in commercially available and industrial DTC induction motor drives [44]. The on-line rotor flux-based speed estimation system calculates the instantaneous rotor speed through the use of equation (32) [44].

(32) $$\omega_{r}=\omega_{mr}-\omega_{sl}$$

Where $\omega_{mr}$ is the speed of the rotor flux relative to the stator, and $\omega_{sl}$ is the angular slip frequency. The speed of the rotor flux and the angular slip frequency can be found using equations (33) and (34) respectively [44], [45].

(33) $$\omega_{mr}=\frac{\psi_{rd}\frac{d\psi_{rq}}{dt}-\psi_{rq}\frac{d\psi_{rd}}{dt}}{{\left|\psi_{r}\right|}^{2}}$$

(34) $$\omega_{sl}=\frac{2T_{e}R_{r}}{3P{\left|\psi_{r}\right|}^{2}}$$

Where *P* is the number of pole pairs, and $R_{r}$ is the rotor resistance. As a result, using equations (27)-(34), the instantaneous rotor speed can be estimated using equation (35) [44], [45].

(35) $$\omega_{r}=\frac{\psi_{rd}\frac{d\psi_{rq}}{dt}-\psi_{rq}\frac{d\psi_{rd}}{dt}}{{\left|\psi_{r}\right|}^{2}}-\frac{2T_{e}R_{r}}{3P{\left|\psi_{r}\right|}^{2}}$$

All equivalent circuit parameters of the motor used in the on-line speed estimation system are given in Appendix A, Table A2. All calculations required for the estimation of the instantaneous rotor speed must be performed during the operation of the system. As a result, the calculations are required to be implemented in an estimator model.

## 11 Sensorless speed control results

**Figure 30:**
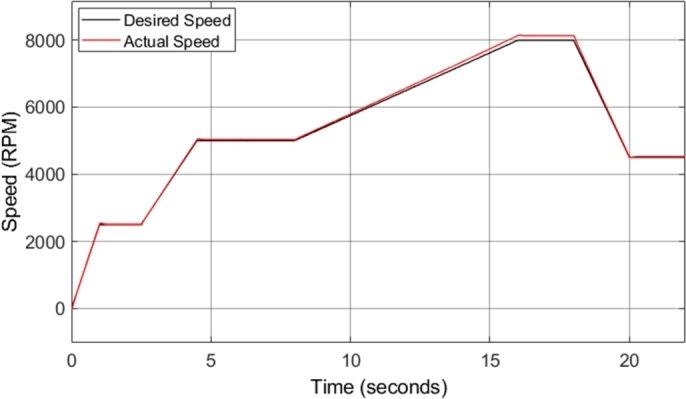
Sensorless DTC - Induction motor speed.

The structure of the DTC-SVM mechanism remains the same when sensorless control is added; however, a dynamic speed estimator is also included in the model, based on equation (35). In addition, the sensorless DTC-SVM model developed in this paper includes field-weakening control, and the necessary torque limits. Fig. 30 shows the motor speed in comparison to the reference or desired speed. The torque load was adjusted to prevent torque limiting, allowing for the speed response obtained with the implementation of sensorless control, under normal operating conditions as well as in the field-weakening region to be easily observed.

**Figure 31:**
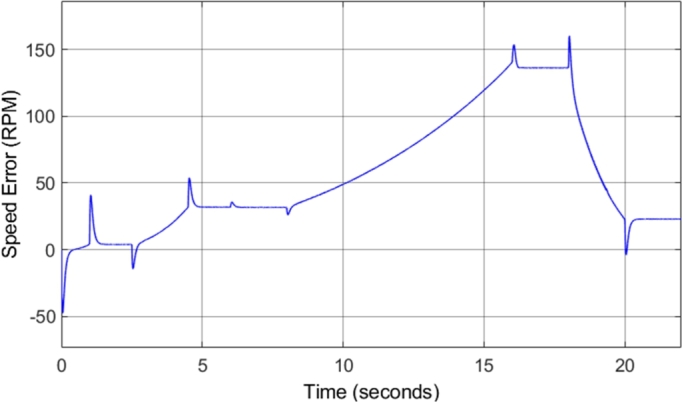
Sensorless DTC - Induction motor speed error.

Fig. 31 shows the induction motor speed error when compared to the desired or reference speed. The results in both Fig. 30 and Fig. 31 indicate that the desired speed is correctly tracked throughout the drive cycle, with very little overshoot or undershoot. However, a small amount of steady-state error can be noticed, which increases with increasing speed. At a desired speed of 8000 r.p.m., a steady-state error of approximately 1.7% can be observed. This is the highest percentage error observed at any point during the drive cycle, and as a result, it can be concluded that the sensorless speed control method implemented has an accuracy of greater than 98% throughout the required speed range simulated. This is a desired result, indicating that the implemented method allows for accurate control of the motor speed. The slight steady-state speed error observed can be attributed to slight ripple and distortion in the parameters used in the speed estimation model, such as the stator currents.

**Figure 32:**
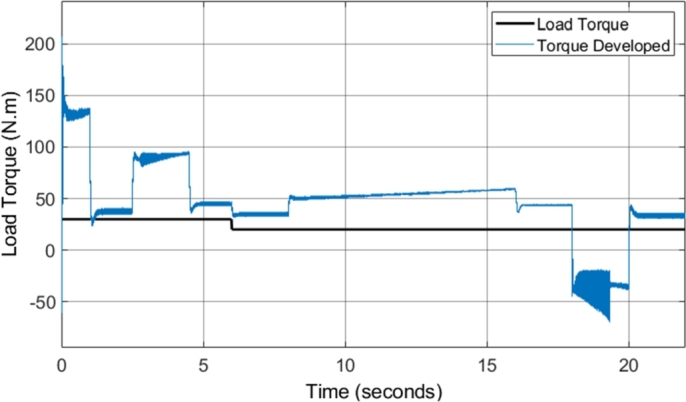
Sensorless DTC - Torque developed by Induction motor (1 MHz).

Fig. 32 shows the torque developed by the induction motor when sensorless DTC-SVM is utilized. The result indicates that the torque ripple is maintained within suitable levels. Torque limiting did not occur due to the reduced torque load utilized in the sensorless DTC-SVM simulation. It can be concluded that the implementation of sensorless control does not have a significant impact on the torque ripple, with only a very small increase in ripple seen in some operating conditions.

**Figure 33:**
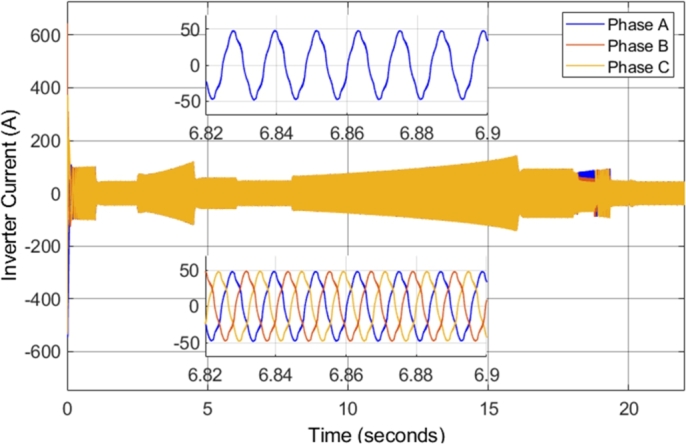
Sensorless DTC - Three-phase inverter current.

The inverter voltages observed in the simulation remained the same as compared to those observed in the DTC-SVM mechanism when sensorless control had not been implemented. Fig. 33 shows the inverter current supplied to the induction motor during the entire drive cycle. In addition, zoomed-in portions of both the Phase A and the three-phase steady-state inverter current supplied to the induction motor, when the motor is operating at 5000 r.p.m. with a load torque of 20 N-m, are shown. The implemented sensorless control causes slight distortion in the current waveform; however, the current ripple is unaffected.

**Figure 34:**
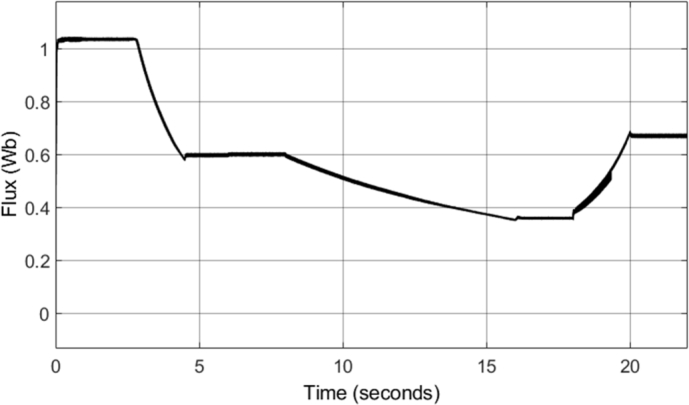
Sensorless DTC – Stator flux magnitude.

**Figure 35:**
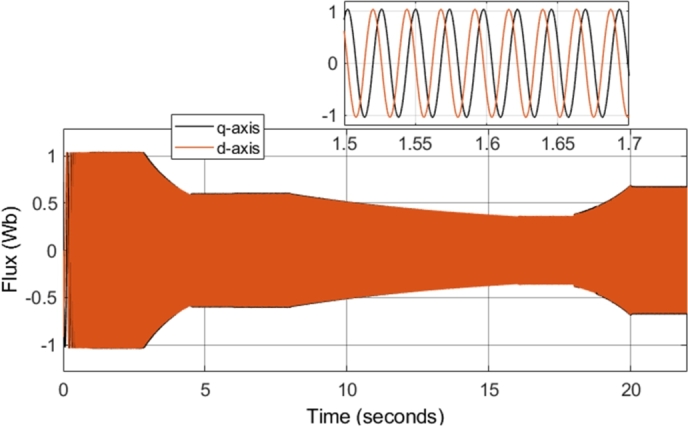
Sensorless DTC - Stator Flux (d- and q-axis).

Fig. 34 shows the stator flux magnitude during the entire drive cycle, and Fig. 35 shows the d- and q- axis components of the stator flux. The results indicate that the stator flux is correctly varied, showing correct field-weakening control of the motor above the specified base speed. The sensorless speed control technique implemented results in a marginally increased flux ripple; however, it does not have a significant effect on the results obtained.

The sensorless DTC-SVM results obtained are a significant advancement in the traction motor drive system implemented. Sensorless control is majorly advantageous in EV systems, enabling reduced hardware complexity, reduced cost, and less maintenance requirements [43]. The implemented algorithm allowed for speed control with an accuracy of greater than 98% across the entire speed range required, with only a very small increase in torque and flux ripples. However, the method implemented requires an ideal integral and is sensitive to variation in the motor parameters, especially at low speeds [45]. As a result, further investigation can be carried out into MRAS and artificial intelligence methods discussed in [45].

## 12 Conclusion

The objective of this paper is to investigate the performance of direct torque control in the traction motor control system of a battery electric vehicle. The paper presented contains a parameter matching stage, and investigation into both conventional direct torque control, as well as direct torque control utilizing a space vector modulation strategy in electric vehicle drives. Investigation of conventional direct torque control indicated that the control mechanism, while producing a fast torque response with low steady-state speed error, exhibited high electromagnetic torque and current ripples, which is undesirable in electric vehicle applications. However, the implementation of improvements to the control mechanism, with the use of direct torque control with a space vector modulation technique showed significantly favorable results. In addition, field-weakening control, and well as sensorless control can also be implemented in conjunction with the system in order to present a complete traction motor control system. The complete control system investigated presented favorable results, and is suitable for use in electric vehicle systems. The results indicated that a fast torque response is achieved, with a perfectly controlled field-weakening mechanism, and sensorless control with accuracy of greater than 98% across the entire speed range. Some steady-state torque error can be noticed, and can be said to be a characteristic of the control scheme investigated. Further investigation can be made into the implementation of different direct torque control systems in the complete traction motor drive mechanism. Other systems include model predictive based direct torque control, sliding mode based direct torque control and deadbeat direct torque control. Simulation of such techniques will be included in later articles as comprehensive investigations are required.

## Declarations

### Author contribution statement

Matthew L. De Klerk & Akshay K. Saha: Conceived and designed the experiments; Performed the experiments; Analyzed and interpreted the data; Contributed reagents, materials, analysis tools or data; Wrote the paper.

### Funding statement

This research did not receive any specific grant from funding agencies in the public, commercial, or not-for-profit sectors.

### Data availability statement

No data was used for the research described in the article

### Declaration of interests statement

The authors declare no conflict of interest.

### Additional information

Supplementary content related to this article has been published online at https://doi.org/10.1016/j.heliyon.2022.e09265.

## Appendix A Supplementary material

mmc1.docx Table A1: Induction motor specifications [47]. Table A2: Induction motor equivalent circuit parameters. mmc1.docx
