# Supplementary material for: Performance analysis of DTC-SVM in a complete traction motor control mechanism for a battery electric vehicle
Source: Heliyon. 2022 Apr 12;8(4):e09265. doi: 10.1016/j.heliyon.2022.e09265 (PMC9026649; doi:10.1016/j.heliyon.2022.e09265)
Supplement: mmc1.docx — Table A1: Induction motor specifications [47]. Table A2: Induction motor equivalent circuit parameters. [file mmc1.docx]

# Appendix A

TABLE A1

INDUCTION MOTOR SPECIFICATIONS [47]

| **Parameter** | **Value** |
| --- | --- |
| Output Power | 37 kW |
| Input Voltage | 400 V 50 Hz |
| Number of Poles | 2 |
| Synchronous Speed | 3000 r.p.m. |
| Rated Speed | 2952 r.p.m. |
| Full Load Efficiency | 92.5% |
| Nominal Current | 63.5 A |
| Rated Torque | 119 N-m |

TABLE A2

INDUCTION MOTOR EQUIVALENT CIRCUIT PARAMETERS

| **Parameter** | **Value** |
| --- | --- |
| Stator Resistance ($R_{s})$ | 0.08233 Ω |
| Stator Leakage Inductance ($L_{ls})$ | 0.000724 H |
| Rotor Resistance ($R_{r})$ | 0.0503 Ω |
| Rotor Leakage Inductance ($L_{lr})$ | 0.000724 H |
| Mutual Inductance ($L_{m})$ | 0.02711 H |
| Stator Self-Inductance ($L_{s})$ | 0.027834 H |
| Rotor Self-Inductance ($L_{r})$ | 0.027834 H |
| Inertia | 0.37 kg.m^2^ |
| Frication Factor | 0.02791 N-m.s |
